# Association of single-nucleotide polymorphisms in *NAT9 *and *MAP3K3* genes with litter size traits in Berkshire pigs

**DOI:** 10.5194/aab-61-379-2018

**Published:** 2018-10-02

**Authors:** Jung Hye Hwang, Sang Mi An, Go Eun Yu, Da Hye Park, Deok Gyeong Kang, Tae Wan Kim, Hwa Chun Park, Jeongim Ha, Chul Wook Kim

**Affiliations:** 1 Swine Science and Technology Center, Gyeongnam National University of Science & Technology, Jinju 660-758, South Korea; 2 Dasan Pig Breeding Co., San 64-2, Gasan-ri, Eunbong-eub, Namwon-si 590-831, South Korea; * These authors contributed equally to this work

## Abstract

Litter size is an economically important trait in the pig industry. We aimed to identify genetic markers associated with litter size, which can be used in breeding programs for improving reproductive traits. Single-nucleotide polymorphisms (SNPs) of Berkshire pigs in the *N-acetyltransferase 9 *(*NAT9*) and *Mitogen-activated protein kinase kinase kinase 3 (MAP3K3)* genes were from RNA sequencing results, and already exist in the databank (NCBI), and were confirmed by polymerase chain reaction and restriction fragment length polymorphism (PCR-RFLP). A total of 272 Berkshire sows were used to examine the genotype, and their association with litter size traits was analyzed. The *NAT9* SNP was located in chromosome 12 exon 640 mRNA (A > G) and the *MAP3K3* SNP was located in chromosome 12 intron 11 (80, C > T). Association analysis indicated that the GG genotype of *NAT9* and the CT genotype of *MAP3K3* had the highest values for litter size traits. The GG genotype expressed higher levels of *NAT9* mRNA in the endometrium than the other genotypes did, and a positive correlation was found between litter size traits and *NAT9*, but not *MAP3K3* expression level. These results indicate that the *NAT9* and *MAP3K3* can be used as candidate genes applicable in breeding program for the improvement of litter size traits in Berkshire pigs.

## 1 Introduction

Litter size is an economically important trait in the pig industry. Increasing litter size is of great interest to the pig industry (Quinton et al., 2006). However, reproductive traits have low heritability, and improvement of these traits occur at a low rate and efficiency (Okere and Nelson, 2002). Optimized breeding programs are hard to establish because litter size traits can be affected by season, parity, housing, and feeding conditions (Distl, 2007; Knecht et al., 2015). Therefore, various efforts have been made to develop genetic markers affecting litter size (Drogemuller et al., 2001; Coster et al., 2012; Minghua et al., 2015; An et al., 2017).

Berkshire pigs have excellent meat quality in terms of marbling, juiciness, tenderness, and flavor, and meat of this breed is the most popular in South Korea and Japan (Lee et al., 2015). However, Berkshire pigs have smaller litter sizes than the other breeds have (McMullen, 2006). Thus, in order to improve the income of farmers, it is necessary to develop genetic markers for the improvement of litter size traits in addition to the improvement of environmental conditions.

N-acetyltransferase is an enzyme that catalyzes the transfer of acetyl groups from acetyl-CoA to arylamines, via an acetyl histidine intermediate (Riddle and Jencks, 1971; Ma et al., 2012). *N-acetyltransferase 9* (*NAT9*) functions in N-acetyltransferase activity. The *NAT9* gene is a new member of the N-acetyltransferase family (Helms et al., 2003). Previous studies reported that *NAT9* plays an important role in embryonic brain development, and that it might be important for adult brain and gonad function (Zou et al., 2006). However, few studies have reported an association of the *NAT9* gene with litter size in pigs.

*Mitogen-activated protein kinase kinase kinase 3 (MAP3K3)*, also known as *MEKK3*, is a member of the mitogen-activated protein kinase (MAPK) family. *MEKK3* is a *MAP3K3* that regulates extracellular signal-regulated kinase (ERK), p38, and c-Jun N-terminal kinases (JNK) MAP kinases (Fritz et al., 2006; Craig et al., 2008). The ERK signaling pathway participates in the maintenance of normal pregnancy (Wang et al., 2016) and plays an important role in the regulation of uterine arterial contractility (Xiao and Zhang, 2002). In addition, the pregnancy-associated risk factors can cause oxidative-stress-induced p38 MAPK activation, leading to senescence and premature aging of fetal tissues (Menon and Papaconstantinou, 2016). Deletion of *MEKK3* in the endocardium leads to embryonic death, likely due to a decreased myocardium (Rawnsley, 2013). In general, regulators of angiogenesis are known to regulate vascular endothelial growth factor (VEGF) and placental growth factor (PGF) (Zygmunt et al., 2003). VEGF signaling plays an important role in the development and maintenance of the placental vascular function during pregnancy (Cheung, 1997). Moreover, in pigs, it is reported that mRNA of VEGF is significantly increased in the endometrium during early pregnancy (Welter et al., 2003).

In the present study, we aimed to identify the single-nucleotide polymorphisms (SNPs) in these two genes and elucidate the significant associations with reproductive traits.

## 2 Materials and methods

### 2.1 Ethics statement

The experimental protocols of this study have been approved by the Animal Care and Use Committee of Gyeongnam National University of Science and Technology Institution (2016-9).

### 2.2 Animals

To detect SNPs in the *NAT9* and *MAP3K3* genes, we performed RNA sequencing (RNA-Seq). RNA was isolated from endometrial tissue of Berkshire pigs by using the RNA-Seq sample preparation kit (Illumina, San Diego, CA, USA). A total of 272 Berkshire sows were reared under the same environmental conditions (Dasan Pig Breeding Co., Namwon, South Korea). Genomic DNA from the blood of 272 Berkshire pigs was isolated using the Wizard Genomic DNA Purification Kit (Promega, Madison, WI, USA). The primary experiment was performed using 140 pigs for the *NAT9* gene and 139 pigs for the *MAP3K3* gene. The secondary experiment was conducted with 132 Berkshire pigs for the *NAT9* gene and 133 Berkshire pigs for the *MAP3K3* gene. In order to determine the expression level in endometrial tissue of the different genotypes, tissue from the middle portion of each uterine horn of all the sows studied was harvested at the time of slaughter and rapidly frozen in liquid nitrogen. In addition, we investigated the expression profile of mRNA in various tissues. Total RNA was isolated from the liver, stomach, lung, kidney, large intestine, small intestine, spleen, muscle, and endometrium (n=3). RNA was isolated using TRIzol reagent (Molecular Research Center, Cincinnati, OH, USA) according to the manufacturer's protocol. The quality of isolated RNA was evaluated with a spectrophotometer (A${}_{260}$/A${}_{280}$; ND-1000; NanoDrop Technologies, Wilmington, DE, USA). The Superscript II (Invitrogen, Carlsbad, CA, USA) was used to synthesize cDNA in a total reaction volume of 20 µL.

### 2.3 SNP detection by RNA-Seq

RNA-Seq was performed with isolated RNA as previously described by Jung et al. (2012). RNA variations were identified based on the pig genome database using the RNA-DNA difference method (An et al., 2017). The SNPs were detected by assembling and mapping raw data to UniGene (https://www.ncbi.nlm.nih.gov/unigene, last access: August 2017), and the information of the detected SNPs obtained using the dbSNP database of NCBI 0 (rs55620935 for *MAP3K3* and rs334957540 for *NAT9*).

### 2.4 Analysis of *NAT9* and *MAP3K3 *genotypes

A PCR-RFLP method was used to analyze the SNP genotypes of the *NAT9 *and *MAP3K3 *genes. The PCR was performed in a 15 µL volume containing 50 ng genomic DNA (1 µL), 1.5 µL of 10× Taq DNA polymerase buffer, 1.2 µL of 2.5 mM mixed dNTPs, 1.0 µL of each forward and reverse primer (*NAT9*, 5${}^{\prime }$-CGC GCT CTC TGG CTT CCG CA-3${}^{\prime }$ and 5${}^{\prime }$-CCC TCA ACT CTG GCT CCA CG-3${}^{\prime }$; *MAP3K3*, 5${}^{\prime }$-TCA AGG CAA CCT GTT CAC CC-3${}^{\prime }$ and 5${}^{\prime }$-AGC ACA TCT CAG CAC AGC AG-3${}^{\prime }$), 0.1 µL e-Taq DNA polymerase, and 9.2 µL ${\mathrm{dH}}_{2}O$. The PCR amplification was performed on a GeneAmp PCR System 9700 (Applied Biosystems, Foster City, CA, USA). For *NAT9*, PCR amplification was performed using 35 cycles of 30 s at 94 ${}^{\circ }$C, 30 s at 62 ${}^{\circ }$C, and 30 s at 72 ${}^{\circ }$C. For *MAP3K3*, PCR cycles were carried out for 30 s at 94 ${}^{\circ }$C, 30 s at 65 ${}^{\circ }$C, and 30 s at 72 ${}^{\circ }$C. The PCR products were digested with *Alu*I (New England Biolabs, Ipswich, MA, USA) and *Xcm*I for 12 h at 37 ${}^{\circ }$C to analyze the genotype of *NAT9* and *MAP3K3*, respectively. The products of digestion were resolved on 2.5 % and 4 % agarose gel for the detection of alleles. The A allele of *NAT9* was indicated by cleavage into two products (159 and 295 bp), whereas the G allele was not cleaved (454 bp). The C allele of the* MAP3K3* gene was not cleaved (600 bp), whereas the T allele was cleaved into two products (240 and 360 bp).

### 2.5 Analysis of litter size traits

The analytical model used to estimate the genetic parameters and estimated breeding value (EBV) were as follows:

1 $$y_{tijkl}={µ}_{t}+l_{ti}+f_{tj}+a_{tk}+p_{tk}+e_{tijkl},$$

where $y_{tijkl}$ is the t-th observed value of the total number of piglets born (TNB) and number of pigs born alive (NBA), µ${}_{t}$ is the average of the t-th trait, $l_{ti}$ is the fixed effect of the i-th parity of the t-th reproductive trait, $f_{tj}$ is the fixed effect of the j-th delivery week of the t-th trait, $a_{tk}$ is the k-th individual breeding value of the t-th trait, $p_{tk}$ is the k-th individual permanent environmental effect of the t-th trait, and $e_{tijkl}$ is random error of the t-th trait. In addition, Var(a)=A⋅σ2aVar(p)=I⋅σ2p, and Var(e)=I⋅σ2e, where A is the coefficient of the relationship matrix of individual additive genetic relationships and I is the identity matrix applied to the linear model.

### 2.6 RNA extraction and reverse transcription PCR

Next we evaluated the variation in mRNA expression level of *NAT9* and *MAP3K3* among genotypes to analyze the relationship between the mRNA level and litter size traits. Reverse transcription (RT)-PCR was performed using gene-specific primers, which were designed using Primer 3 (http://primer3.ut.ee/, last access: August 2017). The primer sequences for *NAT9* were as follows: forward 5${}^{\prime }$-TCT GAC CAC GTT TGA GGC TA-3${}^{\prime }$ and reverse 5${}^{\prime }$-CCT GGT CTT ACA CTC CCG TT-3${}^{\prime }$; and those for *MAP3K3* were as follows: forward 5${}^{\prime }$-TGA ACA GCC CCA CAG TAA CA-3${}^{\prime }$ and reverse 5${}^{\prime }$-ATG TAG ATC CAG AGG CTG CC-3${}^{\prime }$. The primer sequences for amplification of peptidylprolyl isomerase A (PPIA), which was used as an internal control, were as follows: forward 5${}^{\prime }$-CAC AAA CGG TTC CCA GTT TT-3${}^{\prime }$ and reverse 5${}^{\prime }$-TGT CCA CAG TCA GCA ATG GT-3${}^{\prime }$. The reaction conditions for *NAT9* and *MAP3K3* were 30 and 35 cycles, respectively, at 94 ${}^{\circ }$C for 20 s, 60 ${}^{\circ }$C for 20 s, and 72 ${}^{\circ }$C for 20 s. The products were electrophoresed on a 2 % agarose gel. Band intensity was analyzed using ImageJ (National Institutes of Health, Bethesda, MD, USA). The fold change in mRNA expression level was calculated by comparing the expression level in a specific genotype with the lowest expression level in the other groups.

### 2.7 Statistical analysis

**Table 1:** Identification of SNPs in *NAT9.*

Gene name (accession number)	*NAT9* (GU373685)
Chromosome number	Chr. 12
SNP position	mRNA 640
Reference sequence	A
Variant sequence	G

**Table 2:** Allele frequency of the SNP in *NAT9.*

Gene	Allele		${}^{a}$MAF	${}^{b}$HWE
	Major	Minor		
NAT9	A	G	0.2571	0.5781
	0.743	0.257		

${}^{a}$MAF: a minor allele frequency. ${}^{b}$HWE: permutation-based chi-square test for Hardy–Weinberg equilibrium.

SAS v.9.1 (SAS Institute, Cary, NC, USA) was used to analyze correlations of the genotypes of *NAT9* and *MAP3K3* with litter size traits. The significance of differences between genotype frequencies of traits was confirmed with the Mann–Whitney and Student's t test, and by analysis of variance (ANOVA) with the Kruskal–Wallis test (p<0.01 and <0.05). ANOVA results were further analyzed with the Bonferroni test. The RT-PCR results were analyzed using SPSS software (IBM Corp, Armonk, NY, USA). Statistical significance was considered at p<0.05.

## 3 Results

### 3.1 Identification of SNP in the *NAT9* gene and association of the *NAT9* genotype with litter size traits

An SNP was identified in the *NAT9* gene (mRNA 640, A > G; rs334957540) using RNA-Seq (Table 1). The major and minor alleles were A and G, respectively. MAF and Hardy–Weinberg equilibrium (HWE) values were suitable for SNP analysis (Table 2). The genotype of the SNP in the *NAT9* gene was significantly associated with breeding value based on TNB (BVT; p<0.05). The GG genotype had higher BVT values than the other genotypes (Table 3). Repetition of the experiment confirmed the significant association of the SNP in the *NAT9* gene with litter size traits. Pigs with the genotype GG showed significantly higher values of TNB and BVT than those of the other genotypes, and that was consistent with the results obtained in the primary experiment. The GG genotype showed the highest NBA value among genotypes although the difference was not significant (Table 4).

**Table 3:** Association of litter size traits with *NAT9* genotypes in the primary experiment.

Gene		*NAT9*			p value
Genotype		AA	GA	GG	
		(76)	(56)	(8)	
Traits	TNB${}^{a}$	8.266±2.532	8.371±2.253	10.165±1.256	0.1006
	NBA${}^{b}$	7.402±2.232	7.376±2.006	8.736±1.419	0.2183
	BVT${}^{c}$	$0.041\pm 0.826^{*}$	$0.042\pm 0.793^{*}$	$0.855\pm 0.163^{*}$	0.0209

${}^{*}$Significant differences among each genotype group (p<0.05). ${}^{a}$TNB: total number born. ${}^{b}$NBA: Number of pigs born alive. ${}^{c}$BVT: breeding value based on TNB.

### 3.2 *NAT9* mRNA expression analysis according to its genotype

**Figure 1:**
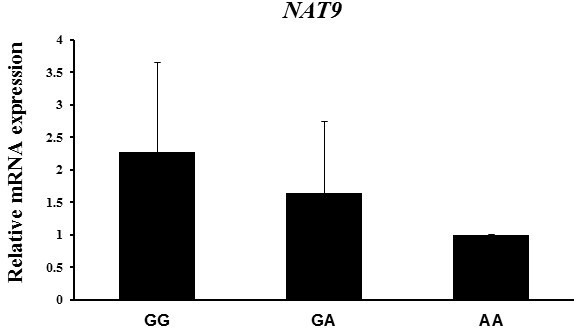
*NAT9* mRNA expression in endometrial tissues according to genotype, as determined by RT-PCR. Peptidylprolyl isomerase A (PPIA) was used as an internal control. The fold change in mRNA expression level was calculated by comparing the mRNA expression level in the genotype with the lowest expression with that of the other groups. Data represent mean ± SD.

SNPs in the 3${}^{\prime }$-UTR (untranslated region) of *NAT9* have been known to regulate *NAT9* mRNA expression (Chen et al., 2016). Next we determined whether mRNA expression of *NAT9* varied among genotypes in endometrial tissue samples, because the SNP of the *NAT9* gene was located in its 3${}^{\prime }$-UTR. We found that mRNA of *NAT9* was closely associated with litter size traits. Consequently, the *NAT9* mRNA level was higher in the GG genotype than in the AA genotype, and the highest value of the reproductive traits was found in the former, although no correlation between the three genotypes was found (Fig. 1).

### 3.3 Identification of SNP in the *MAP3K3* gene and analysis of association between the* MAP3K3* genotype and litter size traits

**Table 4:** Association of litter size traits with *NAT9* genotypes in the secondary experiment.

Gene		*NAT9*			p value
Genotype		AA	GA	GG	
		(72)	(52)	(8)	
traits	TNB${}^{a}$	$8.759\pm 1.942^{*}$	$8.853\pm 1.754^{*}$	$10.405\pm 1.025^{*}$	0.0298
	NBA${}^{b}$	7.799±1.744	7.642±1.508	8.778±1.255	0.1901
	BVT${}^{c}$	$0.180\pm 0.792^{*}$	$0.082\pm 0.788^{*}$	$0.911\pm 0.169^{*}$	0.0200

${}^{*}$Significant differences among each genotype group (p<0.05). ${}^{a}$TNB: total number born. ${}^{b}$NBA: Number of pigs born alive. ${}^{c}$BVT: breeding value based on TNB.

We identified an SNP within the *MAP3K3* gene by RNA-Seq of endometrial tissues. The *MAP3K3* SNP was located in intron 11 (rs55620935, Table 5). The major allele was C, whereas the minor allele was T (Table 5). The HWE and MAF values indicated that the alleles were suitable for SNP analysis (Table 6). We found that the SNP in the *MAP3K3* gene was significantly associated with TNB (p<0.01), NBA (p<0.05), and BVT (p<0.05). The CT genotype had the highest values of all the traits (Table 7). To confirm the association detected, we repeated the experiment with pigs reared in the same farm with other slaughter batches. Although the association of the SNP in *MAP3K3* with litter size traits in experimental pigs from other farms was not significant, the effect was consistent with the results obtained in the primary experiment. Nonetheless, CT was associated with the highest TNB, NBA, and BVT values among the genotypes examined (Table 8).

**Table 5:** Identification of SNPs in *MAP3K3.*

Gene name (accession number)	*MAP3K3 *(AK236570)
Chromosome number	Chr. 12
SNP position	Intron 11, 80
Reference sequence	C
Variant sequence	T

### 3.4 *MAP3K3* mRNA expression according to genotype

**Figure 2:**
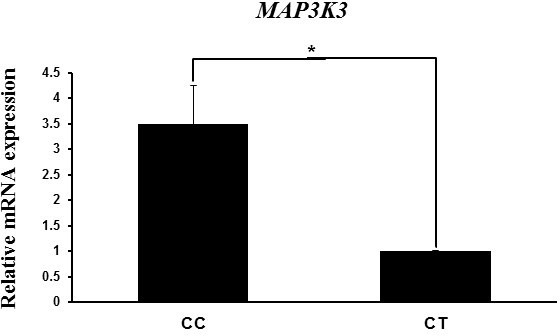
*MAP3K3* mRNA expression in endometrial tissues according to genotype, as determined by RT-PCR. Peptidylprolyl isomerase A (PPIA) was used as an internal control. The fold change in mRNA expression level was calculated by comparing the mRNA expression level in the genotype with the lowest expression with that the other groups. Data represent mean ± SD. ${}^{*}$ p<0.05.

Next we evaluated the variation in *MAP3K3* mRNA expression level with genotype to analyze the relationship of the mRNA level with litter size traits. *MAP3K3* mRNA expression level in the endometrial tissues of Berkshire pigs was higher in the CC genotype than in the CT genotype (Fig. 2). Although the values of the litter size traits were higher in the CT genotype than in the CC genotype, the mRNA expression level showed the opposite trend. These results indicate a negative correlation between *MAP3K3* mRNA expression and litter size traits.

## 4 Discussion

Litter size traits of importance to the pig industry are affected by many environmental and genetic factors (Okere and Nelson, 2002; Quinton et al., 2006; Distl, 2007; Knecht et al., 2015). The present study aimed to identify molecular markers that can be applied for breeding in order to improve litter size in Berkshire pigs. Although Berkshire pigs are the most popular with consumers owing to their excellent meat quality, this breed has a smaller litter size than that of the other breeds (Suzuki et al., 2003; Lee et al., 2015). Genetic markers have been developed for improving the litter size traits of Berkshire pigs in a previous study, and the SNPs in *IGFBP2* and *IGFBP3* are linked to litter size traits in Berkshire pigs (An et al., 2017). However, the previous studies to date have focused on the genetic markers of the Landrace and Large White pig breeds (Kumchoo and Mekchay, 2015; Sell-Kubiak et al., 2015), and the studies on Berkshire pigs are relatively less studied. In the present study, we found that genetic variations in *NAT9* and *MAP3K3* are associated with litter size traits in Berkshire pigs.

**Figure 3:**
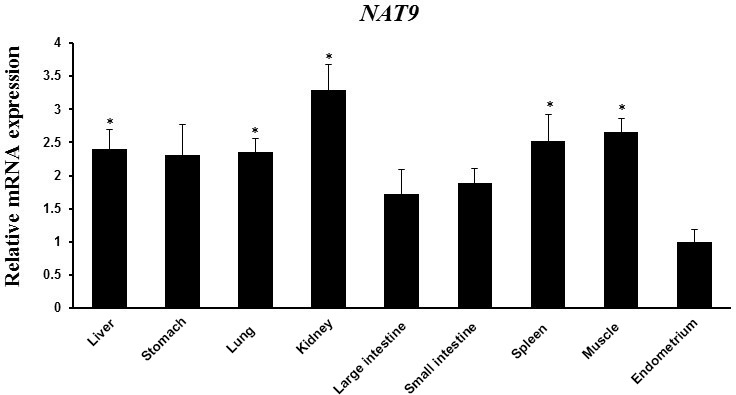
*NAT9* mRNA expression in various tissues (liver, stomach, lung, kidney, large intestine, small intestine, spleen, muscle, and endometrium) as determined by RT-PCR. Peptidylprolyl isomerase A (PPIA) was used as an internal control. The fold change in mRNA expression level was calculated by comparing the lowest expression level in the endometrium with that in the other tissues. Data represent mean ± SD. ${}^{*}$ represents p<0.05.

N-acetyltransferase is expressed in pre-implantation embryonic stem cells and may play an important role in folate catabolism (Payton et al., 1999). Previous studies suggested that pregnancy results in a greater rate of folate catabolism, which progressively increases during pregnancy, reaching maximum values at the time of maximal fetal growth (McNulty et al., 1993; Higgins et al., 2000). Thus, the *NAT9* gene is expected to have a similar function, and it might affect litter size. The SNP located in the 3${}^{\prime }$-UTR regions of genes modulates mRNA stability owing to its effects on polyadenylation and regulatory protein–mRNA and miRNA–mRNA interactions (Skeeles et al., 2013). Because the SNP in *NAT9* identified in the present study was located in its 3${}^{\prime }$-UTR, it could be predicted to affect mRNA expression and regulate the role of *NAT9* in reproduction. We found that pigs with the GG genotype of *NAT9 *had higher BVT values. In addition, we identified the relationship between expression of *NAT9* mRNA and litter size traits. Pigs with the GG genotype of *NAT9* had elevated levels of mRNA expression and the highest value for the litter size traits. An SNP in *NAT9* at the same position as that identified in the present study was previously linked to the TNB and NBA in Landrace and Large White pigs (Jiugang et al., 2012). Although the pigs with genotype GG had higher TNB and BVT values, Jiugang et al. (2012) reported that pigs with the AA genotype had higher TNB and NBA values. These different results from the same SNP in the *NAT9* gene might be caused by the variation among breeds. The mRNA expression of *NAT9* in spleen, muscle, liver, kidney, and ovary of Large White pigs was reported previously (Jiugang et al., 2012). We found that the mRNA expression of *NAT9* was significantly upregulated in the liver, lung, kidney, spleen, and muscle than in endometrial tissues of Berkshire pigs (Fig. 3). Results of *NAT9* gene expression analysis in different tissues revealed differential expression patterns, which implied that the biological activities of *NAT9* vary among tissues. These findings suggest that *NAT9* positively regulates reproduction. Therefore, it appears that the SNP detected in *NAT9* could be a useful biomarker for improvement of litter size.

**Table 6:** Allele frequency of the SNP in *MAP3K3.*

Gene	Allele		${}^{a}$MAF	${}^{b}$HWE
	Major	Minor		
*MAP3K3*	C	T	0.1367	0.2510
	0.863	0.137		

${}^{a}$MAF: minor allele frequency. ${}^{b}$HWE: permutation-based chi-square test for Hardy–Weinberg equilibrium.

**Table 7:** Association of litter size traits with genotypes of *MAP3K3* in the primary experiment.

Gene		*MAP3K3*			p value
Genotype		CC	CT	TT	
		(102)	(36)	(1)	
Traits	TNB${}^{a}$	$8.059\pm 2.469^{**}$	$9.683\pm 1.990^{**}$	$7.290^{**}$	0.0021
	NBA${}^{b}$	$7.174\pm 2.226^{*}$	$8.381\pm 1.605^{*}$	$6.710^{*}$	0.0125
	BVT${}^{c}$	$-0.001\pm 0.808^{*}$	$0.421\pm 0.732^{*}$	$-0.490^{*}$	0.0189

Significant differences among each genotype group are indicated as ${}^{*}$ (p<0.05), and ${}^{**}$ (p<0.01). ${}^{a}$TNB: Total number born. ${}^{b}$NBA: number of pigs born alive. ${}^{c}$BVT: breeding value based on TNB.

**Table 8:** Association of litter size traits with *MAP3K3* genotypes in the secondary experiment.

Gene		*MAP3K3*			p value
Genotype		CC	CT	TT	
		(96)	(36)	(1)	
Traits	TNB${}^{a}$	8.632±1.973	9.264±1.700	7.290±.	0.1760
	NBA${}^{b}$	7.696±1.756	8.189±1.440	6.710±.	0.2624
	BVT${}^{c}$	0.117±0.828	0.396±0.679	-0.490±.	0.1391

${}^{a}$TNB: total number born. ${}^{b}$NBA: number of pigs born alive. ${}^{c}$BVT: breeding value based on TNB.

**Figure 4:**
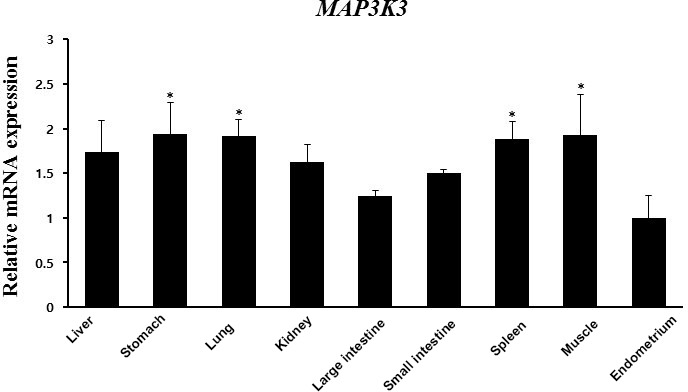
*MAP3K3* mRNA expression in various tissues (liver, stomach, lung, kidney, large intestine, small intestine, spleen, muscle, and endometrium) as determined by RT-PCR. Peptidylprolyl isomerase A (PPIA) was used as an internal control. The fold change in mRNA expression level was calculated by comparing the lowest expression level in the endometrium with that in the other tissues. Data represent mean ± SD. ${}^{*}$ represents p<0.05.

*MAP3K3* is essential for the regulation of extracellular stimulation of cellular responses, such as cell differentiation, proliferation, and apoptosis (Craig et al., 2008). *MAP3K3* directly regulates the stress-activated protein kinase (SAPK) and extracellular signal-regulated protein kinase (ERK) pathways by activating SAPK kinase (SEK) and MAPK/ERK kinase (MEK1/2), respectively, and it is required for p38 activation (Ellinger-Ziegelbauer et al., 1997; Deng et al., 2007). *MAP3K3* is essential for embryonic angiogenesis in mice, and it plays an intrinsic role in embryonic vascular development (Fisher et al., 2015). *MAP3K3* is expressed during embryo development before implantation in mouse (Fong et al., 2007). This suggests that appropriate activation of *MAP3K3* can play important roles in pregnancy. We found that the SNP of *MAP3K3* affected the mRNA expression of the same gene. Pigs with the CT genotype had the largest litter size and the lowest transcript level, which suggests that the expression of *MAP3K3* mRNA can negatively affect litter size. Expression levels of *MAP3K3* were examined in various tissues. *MAP3K3* mRNA expression was increased in the stomach, lung, spleen, and muscle than in endometrial tissues (Fig. 4). The same SNP in the *MAP3K3* gene, which has been identified in the present study, was previously linked to the NBA in German Large White pigs (Spotter et al., 2010). The CT genotype of the *MAP3K3* gene showed an increase in NBA, implying that CT is a more advantageous genotype for breeding.

## 5 Conclusions

In conclusion, we identified SNPs in the *NAT9* and *MAP3K3* genes that are associated with litter size traits in Berkshire pigs. Our results suggest that selecting Berkshire pigs with the GG genotype of *NAT9* and the CT genotype of *MAP3K3 *can produce more piglets. We suggest that these genetic markers would improve litter size in breeding programs, and further studies are necessary to elucidate how these markers can affect litter size and associated mechanisms fully.

## Data Availability

No data sets were used in this article.
